# Strengthening Pathology Capacity to Deliver Quality Cancer Care in Cities in LMICs

**DOI:** 10.1200/GO.20.00604

**Published:** 2021-06-15

**Authors:** Silvina Frech, Luis Eduardo Bravo, Ingrid Rodriguez, Alicia Pomata, Khin Thida Aung, Aung Naing Soe, Beatriz Hornburg, Jeannette Guarner, Jane Brock, Rolando Camacho, Dan Milner

**Affiliations:** ^1^ City Cancer Challenge, Geneva, Switzerland; ^2^ Registro Poblacional de Cáncer de Cali, Hospital Universitario del Valle, Universidad del Valle, Cali, Colombia; ^3^ Hospital de Clínicas, Facultad de Ciencias Médicas, Universidad Nacional de Asunción, San Lorenzo, Paraguay; ^4^ Programa Nacional de Control del Cáncer, Instituto Nacional del Cáncer, Ministerio de Salud y Bienestar Social de Paraguay, Asunción, Paraguay; ^5^ Department of Pathology, University of Medicine, Yangon, Myanmar; ^6^ Centro de Diagnósticos Anátomo-Patológicos, Joinville, Brazil; ^7^ Emory University Hospital, Atlanta, GA; ^8^ Brigham and Women's Hospital, Boston, MA; ^9^ American Society for Clinical Pathology, Chicago, IL

## Abstract

Diagnostic pathology services for oncology health systems are essential; yet, surveys, observations, and hard data from across low- and middle-income countries have revealed that these services are almost always lacking adequate quality and often missing completely. The City Cancer Challenge Foundation (C/Can), the American Society for Clinical Pathology, and C/Can partner cities undertook intense analysis of their existing pathology services as part of a year-long assessment process including the specific formation of a pathology-focused team. Internal and external expert assessments identified sustainable solutions adapted to the local context and level of resources and created specific local implementation projects. Through local leadership, capacity development, and collaboration, services were improved city-wide in three cities: Cali, Colombia; Asunción, Paraguay; and Yangon, Myanmar. Common problems identified across cities included deficiencies in personnel training, equipment, reagents, processes, quality, and coordination. Specific solutions included quality training, standard process development and regulation, implementation of new services, and public-private collaboration. As the first cities joining the C/Can initiative, Cali, Asunción, and Yangon demonstrate the success of the approach and the value of local expertise in identifying problems and solutions. The additional value of international partners' expertise created opportunities for growth through mentorship and technical support. Importantly, the power of healthcare programs with strong political support is emphasized.

## INTRODUCTION

Health systems and public health infrastructure in low- and middle-income countries (LMICs) are equipped variably to deal with their growing cancer burden. Pathology is a core cancer service required for quality and timely diagnosis and patient treatment decision making. Pathology is also necessary for accurate cancer registration, which guides evidence-based public policy decision making for cancer control and allocation of resources. Population-based registries are the most informative and correlate best with pathology laboratory data matched to that population.

**CONTEXT**

**Key Objective**
What are the key factors for successfully implementing interventions aimed at strengthening cancer pathology capacity in cities in resource-constrained settings? The City Cancer Challenge Foundation and the American Society for Clinical Pathology share their learnings and experience from project implementation in three cities in low- and middle-income countries.
**Knowledge Generated**
Local leadership, capacity development leveraging local expertise, and multisectoral collaboration are essential factors for improving pathology capacity and quality in low- and middle-income countries. The value of international partnerships for technical cooperation accompanied by local political commitment is emphasized in the paper.
**Relevance**
Pathologists need to lead the process of identifying gaps in quality pathology service delivery and develop their own local solutions according to the level of resources available.

From a global view, there are major challenges across the cancer care spectrum depending on location, populations, and environment; however, the most common challenges for diagnostics of cancer include the increased needs for patient access, highly trained personnel, consistent equipment performance and maintenance, and stable reagent and supply logistics.^1^ All these factors are driven by fiscal incentives to provide the service, which are often lacking. The roadmap to achieving high-quality patient care for cancer begins with political and social commitment to identifying, diagnosing, and treating cancer.^2^ With such a commitment, the key pillars for a diagnostic program can be put in place including funding, collaborations, training, procurement, and advocacy. Some specific features of diagnostic pathology that must be viewed as crucial and integral to appropriate cancer care include cytology utilization, standardized collection procedures, adequate preservation for the indicated sample type, specimen transport networks, high-quality histology services (including immunohistochemistry [IHC]), and secondary consultation referral pathways for both education and quality control.^3^ Services such as flow cytometry and molecular diagnostics, which are standard of care in high-income countries, are also needed in LMICs but stand at a lower priority until access to medications largely directed by these services are more readily available.

Although in high-income countries, laboratory testing performed for cancer screening typically consumes more funding than laboratory testing for cancer diagnosis, LMICs, which often lack screening programs, will apportion the majority of their pathology budgets initially to cancer diagnostics as they build their programs; therefore, budget considerations for the operation of a pathology laboratory should be based on realistic projections of population need with appropriate numbers and placement of laboratories. Costs of diagnostic services for cancer should be an integrated part of the total budget consideration of cancer care with fee structure and/or pay structure contributing to system viability without creating barriers for patient access. Highly efficient, well-placed laboratories can keep costs per patient low while producing excellent quality and value. Fragmented systems or ad hoc, misaligned systems, which often combine government funded programs with competitive private facilities, create artificial quality dichotomies that lead to poor patient outcomes.

The core principle of the City Cancer Challenge Foundation (C/Can) approach is that cities can drive impact at the national level by crafting local data-driven solutions with the support of a network of global, regional, and local partners that reflect an understanding of the unique local context. This principle was tested and validated in the area of pathology in the first three key learning cities using a uniform strategy based on three basic components: local experts craft their own solutions tailored to local context and available resources in alignment with international best practices; existing local and regional expertise is leveraged to strengthen local capacities; and a network is developed for knowledge-sharing among cities. The strategy is implemented through a combination of on-site visits, virtual expert knowledge exchanges, remote consultations, and city workshops. The current manuscript describes the strategic approach, main lessons learned, and preliminary results in the area of cancer pathology through the work implemented in the first three C/Can cities: Cali, Colombia, and Asunción, Paraguay, both located in upper-middle income countries in Latin America; and Yangon, a city located in Myanmar, a lower-middle income country in Southeast Asia.

## METHODS

Following the stakeholder engagement phase and convening the City Executive Committee in Cali, Asunción, and Yangon, multi-institutional technical working groups (TWGs) were formed with professionals representing the main private and public pathology laboratories to lead a comprehensive city-wide needs assessment as described in detail in a previous paper in this series.^4^ The pathology TWG was composed of pathologists, laboratory technicians, and laboratory managers, who worked together, with support from C/Can city manager, to collect critical data on the pathology infrastructure and services provided in the city, and identify main challenges. Data and information were collected using the C/Can needs assessment questionnaire.^4^ The data were then presented to the TWG and local stakeholders, who collectively brainstormed and prioritized sustainable solutions aimed at strengthening the quality of pathology diagnosis, according to the level of resources of the city.

Based on the conclusions from the needs assessment and prioritization exercise, the American Society for Clinical Pathology (ASCP) and C/Can coordinated on-site visits of regional and international experts to the major public and private pathology laboratories to assess the existing infrastructure and hold in-person meetings with stakeholders. This step was required in the process because the assessment questionnaire facilitated the identification of focused-problem areas within pathology, but further discussion and expert collaboration was needed to refine the problems to a root cause for feasible solutions. Prioritized solutions identified by the city TWG following needs assessment, on-site laboratory visits by ASCP, and activity planning are summarized in Table 1.

**TABLE 1:**
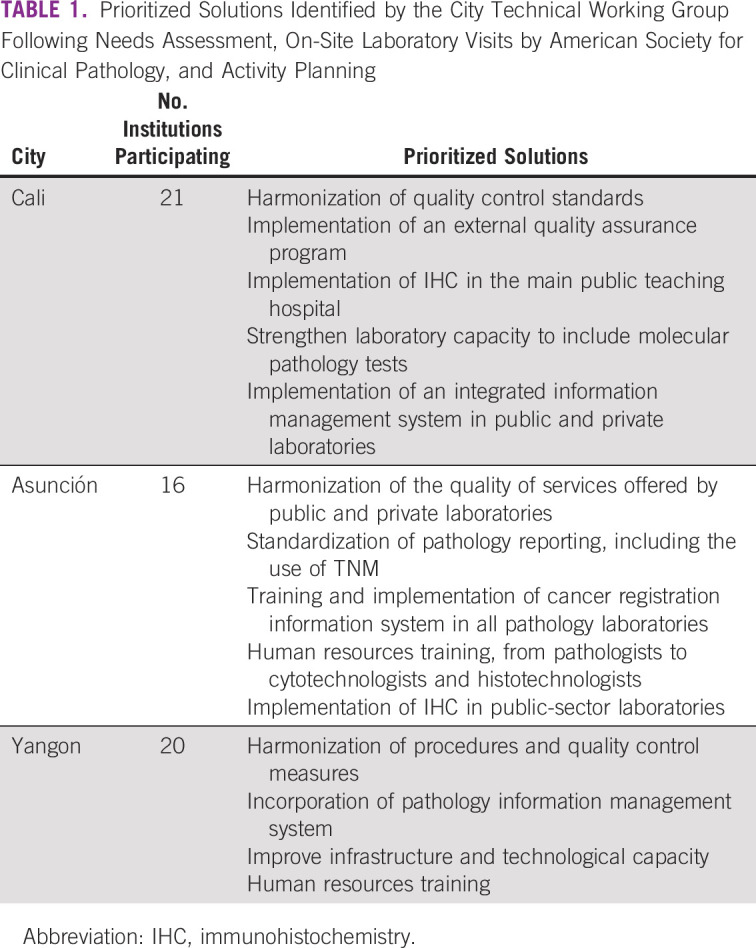
Prioritized Solutions Identified by the City Technical Working Group Following Needs Assessment, On-Site Laboratory Visits by American Society for Clinical Pathology, and Activity Planning

Following ASCP and C/Can recommendations, the pathology TWG agreed on the specific objectives of a project for strengthening the quality of pathology and developed a project plan. A summary of technical assistance and capacity building projects supported by C/Can and ASCP is provided in Table 2. Project management and implementation was coordinated on the ground by C/Can's city manager, with remote support from ASCP experts and C/Can regional and global team. Through a participatory and consensus-based process including review of relevant national and international regulations and adaptation of international quality standards to the local level of resources, key documents or recommendations were developed by the city pathology TWG. Hands-on workshops were delivered to address specific areas of need, such as quality control and laboratory biosafety. Endorsement of the final documents by the C/Can City Executive Committee and submission to local, regional, or national authorities for approval and implementation was the final step in the engagement process directly supported by C/Can. In addition, pathologists are also part of other project teams, such as those working on the development of multidisciplinary teams at the participating institutions and guidelines for the treatment and management of cancer.

**TABLE 2:**
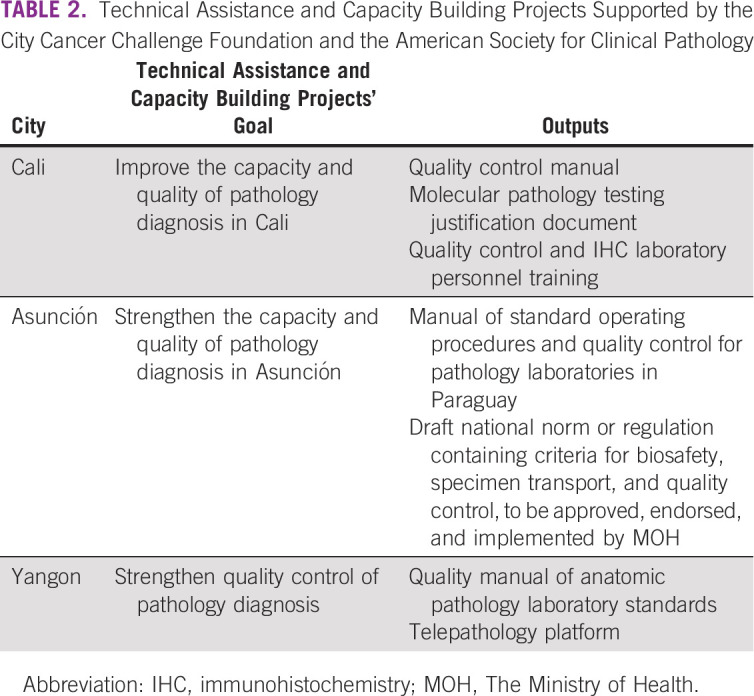
Technical Assistance and Capacity Building Projects Supported by the City Cancer Challenge Foundation and the American Society for Clinical Pathology

Members of the TWG from each city along with selected experts from ASCP and C/Can constructed case studies for each city based on the process described and identified the key priorities, interventions used specifically for pathology, and the lessons learned.

## RESULTS

### Case Study 1—Cali

#### Improving the resources and infrastructure of the main public teaching hospital while strengthening cancer registration

Cali, Colombia, was the first city to join the C/Can initiative. The needs assessment data collection and analysis was carried out by 186 professionals composing the TWGs representing 21 cancer care providers from the public and private sectors, with the guidance of the Cali Population-based Cancer Registry (PBCR) and coordination support from C/Can. The needs assessment was completed from May to December 2017. Project design, development, and implementation took place during 2018 and 2019.

One of the main gaps identified by the Cali pathology TWG was the absence of an IHC service at the Hospital Universitario del Valle (HUV), the main center of the network of public oncological services in the city. IHC is considered a basic requirement for accurate diagnosis and treatment of cancer. As a direct result of C/Can and ASCP efforts to improve quality cancer diagnosis in the city, commitment and leadership from HUV and Universidad del Valle led to the mobilisation of local resources necessary for the implementation of an IHC service at HUV's existing pathology laboratory. Based on the epidemiological cancer profile of Cali prepared by the PBCR, 123 antibodies were selected. The validation process was optimized with tissue microarrays following international quality standards. To date, HUV's IHC new service has processed immunostains in 1,500 cancer cases. Thus, patients at HUV have now access to IHC and improved accuracy of cancer diagnosis.

A second priority identified during the needs assessment was the improvement of the quality of the pathology diagnosis through the development and implementation of a quality control and standard operating procedures manual. As a first step, a workshop “Training in Quality Management for Pathology Laboratories” was organized by C/Can and ASCP with 67 professionals participating from 28 pathology laboratories, including cytology and histology technologists, bacteriologists, and pathologists. Following the workshop, the HUV in collaboration with a private institution, Centro Medico Imbanaco, coordinated the development of a quality control manual, which includes the minimum quality standards for all the pathology laboratories in the city. This manual was reviewed by the ASCP-designated expert. As part of this effort, the College of American Pathologists cancer reporting protocols and an internal quality control program were implemented at the HUV. Since 2020, HUV has participated in an external quality control program by the Royal College of Pathologists of Australasia. Several steps were taken to improve the preanalytical variables, including the change in external providers of quality reagents as well as the establishment of a specific contract for the regular maintenance and control of laboratory equipment. A key success factor has been the appointment of a medical professional dedicated exclusively to the coordination of the quality of the pathology laboratory, focusing on the implementation of standard operating procedures and a pathology laboratory information system. All these efforts and activities have resulted in an improvement in the quality of the processes while ensuring traceability of patient samples.

To meet the increasing patient needs, HUV strengthened its oncology service and, as a result, there was a greater demand for basic histopathology. Qualified human resources were hired by HUV for the pathology laboratory, including four pathologists with training in breast pathology, hematopathology, neuropathology, dermatopathology, and flow cytometry; one histotechnologist for the IHC service; and one laboratory technician to support the three existing histotechnologists. As a result, there was an increase from 4,715 tests carried out in 2018 to 7,892 tests performed in 2019 at the HUV pathology laboratory. The turnaround time has decreased from 2 weeks in 2018, before C/Can and ASCP support, to 8 days.

In parallel, and as a first step toward the goal of improving and integrating information systems in Cali, the PBCR has promoted the use of cancer notification systems and supported the creation of hospital cancer registries in four private tertiary-level hospitals with integrated oncology services.^5,6^

### Case Study 2—Asunción

#### Harmonizing quality and procedures in the public-sector and private-sector laboratories while ensuring an enabling policy framework

The comprehensive needs assessment in Asunción was carried out by 180 professionals from 16 public and private institutions providing cancer care services from July to December 2017. The Asunción pathology TWG was integrated by representatives from the Ministry of Health of Paraguay, the National Cancer Control Program, the Paraguayan Society of Pathology, and the main private and public pathology laboratories in the city. This highly committed group of local pathologists led their own assessment of the current state of pathology in their city, identified the main needs, prioritized the solutions, and implemented a project aimed at improving the quality of pathology laboratories across the city and decreasing the fragmentation of the healthcare system in the country. The project's objectives were to harmonize the quality of services offered by public and private laboratories, strengthen the capacity of human resources, and incorporate standardized pathology synoptic reporting. This key step provided a systemic vision of the main needs and opportunities for improvement. Project design, development, and implementation took place during 2018 and 2019.

Following the situation analysis and activity planning, an ASCP-designated expert from the Latin America region conducted technical visits of seven public and private pathology laboratories to assess their capacities and needs. Through a consensus meeting held with the TWG, and with C/Can and ASCP's guidance and support, the visit's findings, needs, and available resources were discussed. The TWG agreed on a project aimed at improving the delivery of timely diagnostic services to patients with cancer in Asunción. The expected outcome of the project was the reduction of turnaround time of tests from 25 to 30 days to a maximum of 10 days, the use of synoptic reporting for cancer cases, and the provision of consumables all year round for the adequate function of the public laboratories. The local pathologists were all involved from the outset of the project, which ensured a sense of local ownership.

The priority project included the development of a manual of standard operating procedures and quality control for pathology laboratories in Paraguay, since there were no previous regulations to harmonize standards across sectors in the country. A project team was assembled with pathologists from public and private laboratories, and a project coordinator was appointed. All pathologists in the country, who are part of the Paraguayan Pathology Society, were invited to participate in the meetings or provide comments on the manual. The second output of the project was a regulation aimed at harmonizing the minimum quality standards required for all, public and private, pathology laboratories in Asunción, and containing criteria for biosafety, specimen transport, and quality control. The unprecedented efforts resulted in the submission of the draft regulation to the Paraguayan Ministry of Health in August 2020 for endorsement and implementation.

The pathology TWG not only worked inclusively across sectors and institutions, but they also acted as advocates with the Ministry of Health to seek approval and implementation of the national regulation. The professional association of pathologists of Paraguay engaged in outreach activities to educate policy makers as to the importance of pathology quality standards and how policies need to develop to support excellence in this area.

A key complementary and indirect result of C/Can's work in Asunción is the development of the first population-based national cancer registry (PBCR), after long-standing support from International Agency for Research on Cancer's Global Initiative for Cancer Registries (GICR) and PAHO. Paraguay was announced in October 2020 as a GICR partner country, reiterating the country's commitment to advance in the establishment of a PBCR. Under the leadership of the Paraguayan Society of Pathologists and the Health Surveillance Directorate of the Ministry of Health, a Ministerial Resolution S.G. 043 was approved on February 2018 enforcing all health establishments to include the patient's identity card number in all requests and reports of laboratory studies for diagnosis or suspicion of cancer.^7^ This Ministerial Resolution is being implemented in public and private laboratories.

In summary, through a combination of face-to-face and virtual hands-on meetings and technical consultations, training workshops, and site visits, institutional capacity and human resource development was strengthened, multidisciplinary team work was fostered, and a balance of interests and participation of public and private institutions was achieved. The key success factors to start and sustain results were the engagement of both pathologists and laboratory technicians and technologists, who actively participated in the quality control trainings provided by ASCP; the development of a good rapport between C/Can team, local pathologists, and ASCP-designated experts, which led to additional knowledge exchanges through on-site and remote presentations on laboratory biosafety and recommendations for quality improvement in pathology laboratories; and the commitment of pathologists and laboratory technicians to improve their knowledge in quality control and biosafety in a continuous fashion, demonstrating their mindset shift. One of the most important achievements was that all the pathologists in Asunción leveraged the unique opportunity that C/Can brought to the city, to work and act together regardless of their institutions or sectors, and to advocate for quality improvement with the government authorities. The remarkable results achieved in the area of pathology were replicated in other areas supported by C/Can in Asunción.

### Case Study 3—Yangon

#### Establishing a telepathology platform for improvement of pathology diagnosis

Twenty public and private hospitals participated in the needs assessment, which resulted in the situation analysis report of cancer care services in the city. The report revealed that Yangon's public network of clinical laboratories lacked uniformity in terms of infrastructure and technological capacity, level of training and qualifications of personnel, harmonization of diagnostic procedures and quality controls, and integrated pathology information management systems. The needs assessment was completed from October to December 2017. Project design, development, and implementation took place during 2018 and 2019.

The Yangon pathology working group, with support from C/Can and ASCP-designated expert, agreed to prioritize a project with the goal of strengthening quality control and standard operational procedures, developing a telepathology platform, and assessing the real needs for upgrading the most important laboratories in the city to be able to provide quality pathology, clinical laboratory, and transfusion services.

Implementation of the pathology project started with a review of the WHO Laboratory Quality Management System in Yangon. A telepathology service was established using an existing free service by ASCP using the MoticFlow platform and a whole-slide imaging device at the University of Medicine.^8-11^

The Yangon C/Can telepathology working group was composed of senior pathologists from both public and private hospitals as well as the medical university, and 20 pathologists designated by ASCP covering all areas of expertise, coordinated by an experienced telepathology leader. The platform was used initially for a quality assessment of slides produced both in the public and private sectors by medical technologists and junior pathologists, using expert advice from a histotechnologist provided by ASCP. Currently, three to six cases per week are uploaded to the site for expert second opinion, which represents < 1% of the weekly volume of cancer diagnoses. Diagnoses have been rendered in 80% of cases within 72 hours since the implementation of the platform. Experts provide teaching vignettes for all cases and suggest IHC stains if the diagnosis is not definitive. These cases promote academic discussion among Yangon pathologists as many are rare or unusual presentations of disease and not readily diagnosable by general surgical pathologists.

#### Creating a quality manual of anatomic pathology laboratory standards

A working group of 10 pathologists from seven institutions across Yangon created a set of standards for specimen collection and handling, including requisitions, transport, tissue fixation, and accession, as well as standards for intraoperative evaluation, facility safety, personnel skills, gross examination, tissue submission, cassette labeling, tissue processing, embedding, sectioning, slide labeling, tissue disposition, IHC, and in situ hybridization. These standards are the first steps toward writing detailed Standard.

Operating procedures tailored to each individual institution. In addition, the working group created standards for laboratory reporting that include diagnosis, gross, and microscopic description standards, with a focus on using CAP checklists and/or synoptic reports where possible.

In summary, the telepathology platform provided several benefits to the local pathologists including strengthening the local capacities and skills, access to world-class expertise in subspecialty areas of cancer diagnostics, and generated discussions about quality control while reviewing rare and complex cases. Work on a quality manual is well underway, focusing on writing standard operating procedures and establishing laboratory reporting standards that will be adopted across all the government-funded hospitals in Yangon.

The prioritized solutions identified by the city TWG following needs assessment, on-site laboratory visits by ASCP, and activity planning are shown in Table 1. The specific types of technical assistance and capacity building projects supported by C/Can and ASCP in each city are shown in Table 2. A summary of the lessons learned, acknowledged mistakes, and original solutions for each city is provided in Table 3.

**TABLE 3:**
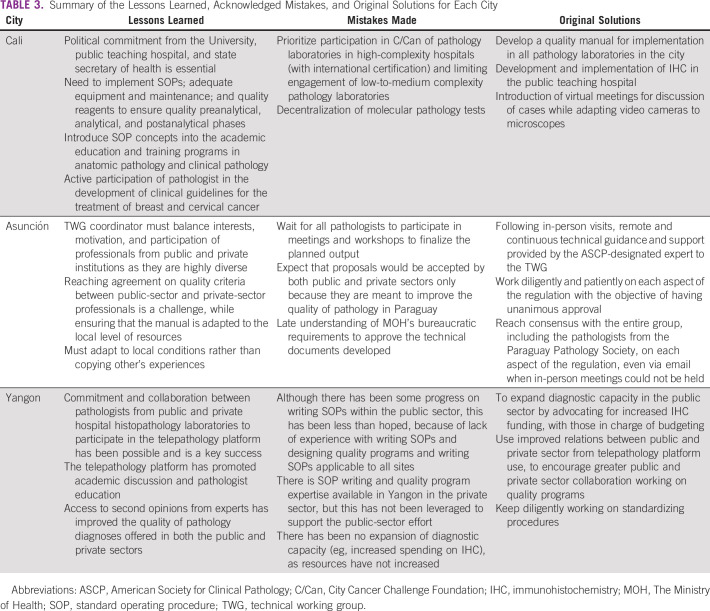
Summary of the Lessons Learned, Acknowledged Mistakes, and Original Solutions for Each City

## DISCUSSION

A key component of the C/Can approach is empowering local city leaders and technical experts to define their own needs and craft sustainable solutions based on the local context and the level of available resources. The adoption and integration of the determined solutions (ie, processes, procedures, and/or regulations) into the local cancer care infrastructure for continued implementation, monitoring, and evaluation by regulatory authorities is essential to ensure sustainability of the interventions and achieving the expected outcomes and impact. Based on the experience of the three cities, several key drivers of success and sustainability of efforts were identified as the necessary elements to be present to effectively and efficiently advance progress toward the long-term goal of strengthening the local capacity and quality of pathology laboratories in LMICs. Each C/Can city has developed a strategic sustainability plan led by local stakeholders, incorporating the key elements described below:

- All stakeholders must work together to develop, refine, and test evidence-based and sustainable solutions adapted to specific setting
- Engaging those responsible for implementation at the design stage of new projects, interventions, or solutions ensures ownership and increases the possibility for its successful implementation and sustainable changes
- Thoughtful identification and convening of the right stakeholders, members, and a coordinator for the pathology working group in each city
- Leadership, commitment to change, and teamwork mindsets are powerful skills that can help overcome health system challenges in LMICs
- Technical support, coordination, and guidance provided by external experts from well-known and respected international organizations brought neutrality to the process and helped maintain a city-wide collective approach while emphasizing the importance of local ownership on the process and results
- Unique and innovative model for professionals from private and public laboratories to work together for the first time in history to assess their own needs and then plan, design, and implement sustainable solutions tailored to the level of resources
- Access to a virtual platform for sharing knowledge and best practices among cities around the world facing similar challenges
- Involvement of trainees and newly graduated pathologists in the quality workshops helped drive innovation as they brought fresh eyes to workflows—going forward, these individuals will be key in continuing with the process improvement cycle
- Involvement of laboratory technicians and technologists in the quality workshops is essential as they are crucial in suggesting and implementing process improvements in their areas of expertise
- The harmonization of quality standards across public and private pathology laboratories should be enforced by the regulatory government agency responsible for ensuring implementation, monitoring, and evaluation of the quality standards in all the pathology laboratories in the cities. This city-wide regulation of good pathology laboratory practices should be complemented with an interpretation manual constructed by the city technical group with engagement of all stakeholders

As part of the eligibility criteria to join C/Can, the first key learning cities highlighted in this manuscript needed to have basic pathology infrastructure in place. Using the lessons learned by C/Can and ASCP's experience with these middle-income countries, we are confident that similar solutions can be developed over time in a variety of settings, and that LMICs will adapt those solutions that suit their environment and level of resources.

One of the intriguing findings of the C/Can city-wide needs assessment was that pathology was identified in all three of the first cities as a high-priority area that needed immediate attention and action. Although this finding is not surprising, identifying such a priority in the setting of all cancer stakeholders including city leaders is a testament to the power and importance of high-quality pathology in a cancer care system.

In conclusion, the initial results of the C/Can approach in the first cities with regards to pathology have been exciting and illuminating. The impact of collaboration and coordination from outside and within pathology demonstrates the value of such collective focus for any aspect of healthcare improvement. Although pathology was prioritized in all three cities, the exact issues, problems identified, and solutions were unique to each city and the result of internal and external experts delving into the problems together to find solutions. The expectation of C/Can and partners is that each new city will have variable results that will always require a deep, expert dive to create value for the city in any new efforts undertaken. Thus, as C/Can grows the program to additional cities, both additional partners in existing areas as well as new partners in new areas of cancer care are paramount to success and strongly encouraged.

To overcome the barriers of moving pathology quality improvement projects to public policy implementation, pathologists must engage as leaders and advocates to move pathology services forward for patients with cancer. Interinstitutional across-sectors groups of pathologists must take ownership of the projects, work inclusively, and leverage opportunities for international technical cooperation. Local pathology associations must engage in outreach activities to educate policy makers as to the importance of pathology quality standards and how policies need to be developed to support excellence in this area.

This innovative approach of bringing together local expertise, international technical assistance, and leadership and advocacy from local pathologists to move technical documents into public policy and practice should be replicated in other cities around the world to improve quality cancer care.
